# Preventive effect of *Terminalia bellirica* (Gaertn.) Roxb. extract on mice infected with *Salmonella Typhimurium*

**DOI:** 10.3389/fcimb.2022.1054205

**Published:** 2023-01-09

**Authors:** Qinghui Kong, Zhenda Shang, Yao Liu, Muhammad Fakhar-e-Alam Kulyar, Sizhu Suo-lang, Yefen Xu, Zhankun Tan, Jiakui Li, Suozhu Liu

**Affiliations:** ^1^ College of Animal Science, Tibet Agricultural and Animal Husbandry University, Nyingchi, China; ^2^ College of Veterinary Medicine, Huazhong Agricultural University, Wuhan, China; ^3^ Tibetan Plateau Feed Processing Engineering Research Center, Nyingchi, China

**Keywords:** *Terminalia bellirica (Gaertn.) Roxb.* extract, mice, *Salmonella Typhimurium*, preventive effect, traditional herbal medicine

## Abstract

*Terminalia bellirica (Gaertn.) Roxb.* (TB) is a traditional herbal combination used in Chinese medicine for the treatment of a broad range of diseases. In this study, thirty KM mice were randomly divided into control (N), infection group (NS), and the TB protection group (HS). Based on its digestive feature, intestinal physical barrier, immunological barrier and gut microbiota effects in vivo on challenged with S.typhimurium mice were investigated after oral administration of 600 mg/kg b.wt of TB for 13 days. The results show that the extract could improve the level of serum immunoglobulins (IgA and IgG), decrease the intestinal cytokine secretion to relieve intestinal cytokine storm, reinforce the intestinal biochemical barrier function by elevating the sIgA expression, and strengthen the intestinal physical barrier function. Simultaneously, based on the V3–V4 region of the 16S rRNA analyzed, the results of the taxonomic structure of the intestinal microbiota demonstrated that the TB prevention effect transformed the key phylotypes of the gut microbiota in *S. Typhimurium-*challenged mice and promoted the multiplication of beneficial bacteria. Furthermore, the abundance of Firmicutes and Deferribacteres increased, while that of Bacteroidetes and Actinobacteria decreased. At the genus level, the abundance of *Ruminococcus* and *Oscillospira* was substantially enhanced, while the other dominant genera showed no significant change between the vehicle control groups and the TB prevention groups. In summary, these results provide evidence that the administration of TB extract can prevent *S. Typhimurium* infection by alleviating the intestinal physical and immunological barriers and normalizing the gut microbiota, highlighting a promising application in clinical treatment. Thus, our results provide new insights into the biological functions of TB for the preventive effect of intestinal inflammation.

## 1. Introduction

Since the first antimicrobials (1911) were developed, the use of antibiotics has dramatically changed human development. Unfortunately, their abuse resulted in serious negative effects, such as drug residue, bacterial resistance, and other problems that challenge food safety and human health. Furthermore, human self-awareness of health has improved, and the addition of antibiotics to feed is banned in the European Union and China (Landers et al., 2012). Therefore, the ongoing search for new veterinary drugs that are antibiotic alternatives is extremely urgent. Notably, traditional Chinese medicines have become dominant in the market, with no drug resistance in animal husbandry (Xia et al., 2022). Traditional Chinese medicine herbs have been prescribed for many conditions, *e*.*g*., improving animal growth performance, immunity, and regulating gut microbiota (Abarike et al., 2019; Wang et al., 2020; Gao et al., 2022). Recent studies indicate that these inhibit the growth of *Staphylococcus aureus*, *Salmonella cholerae suis*, *Escherichia coli*, *Streptococcus agalactiae* (Zou et al., 2022a), *Trichosporon* (Zhang et al., 2020), *Candida glabrata* (Zhang et al., 2021), *Aspergillus flavus*, and *Aspergillus fumigatus* (Xue et al., 2022). Meanwhile, they play a vital role in livestock and poultry farming by enhancing humoral and cellular immune responses (Ma et al., 2013), nutrient digestion and absorption (Kong et al., 2009), and modulating the gut microbiota composition (Waititu et al., 2017). Furthermore, traditional Chinese medicine has the advantages of costing less and being easily prepared.

*Terminalia bellirica* (Gaertn.) Roxb. (TB) is obtained from the fruit of *T. bellirica* tree, which belongs to the Combretaceae family and *Terminalia Lainn*. genus. It is mainly distributed throughout Southeast Asia. A small population, distributed in the Tibet and Yunnan regions of China, use it as folk medicine for diabetes, rheumatism, and hypertension (Tanaka et al., 2018; Chang et al., 2021). In their various extract forms, the fruits elicit multifarious pharmacological properties such as antioxidant, antidiabetic, analgesic, hepatoprotective (Kuriakose et al., 2017), anti-inflammatory (Tanaka et al., 2018), and anti-diarrheal effects (Nigam et al., 2020). Moreover, no signs of toxicity have been observed at concentrations of up to 2,000 mg/kg (Sireeratawong et al., 2013; Jayesh et al, 2017a). Consequently, the current study focuses on evaluating the curative effect of the TB extract against *Salmonella Typhimurium*-induced infection in mice models.

## 2. Materials and methods

### 2.1. Preparation of *T. bellirica (Gaertn.) Roxb.* extract

A total of 100 g of *T. bellirica (Gaertn.) Roxb.* fruits (originating from Yunnan, China) was immersed in 500 ml of deionized water for half an hour. Subsequently, the fruit sample was boiled thrice for 1 h each and filtered. Then, the filtrate was mixed, and the concentration was enriched to 100 ml (final concentration: 1 g/ml). After autoclaving (120°C, 103.4 kPa for 20 min), the water-extracted medicine was stored at −20°C and used up within 30 days. The extraction method was carried out as described by Sireeratawong et al. with minor changes (Sireeratawong et al., 2013). The quality of the extract was confirmed by Lhasa Chinese Lanbaoshi Herbal Medicine Co., Ltd. (Lhasa, China).

### 2.2. Experimental design

The study protocol was approved by the Committee for Animal Research of Tibet Agricultural and Animal Husbandry University, China (unified social credit code: 12540000MB0P013721). Healthy KM (Kunming) female mice (20 ± 2 g) were obtained from the Lhasa Biopharmaceutical Factory (Lhasa, China) and raised in a standard environment (12 h light–dark cycle, 20 ± 2°C, relative humidity: 50 ± 2%) for 1 week. Briefly, the mice were randomly divided into three groups (*n* = 10 per group), *i*.*e*., the vehicle control group (N group), the infection group (NS group), and the TB protection group (HS group). The mice in the N and NS groups were gavaged with deionized water, while the HS group animals received TB at 600 mg/kg b.wt (Sireeratawong et al., 2013; Jayesh et al, 2017a) for 13 consecutive days. Moreover, the NS and HS groups were administered with *S. Typhimurium* (CMCC 50115, 1 × 10^8^ CFU/day) for 3 days. Food was withheld for 3 h after administering the extracts. Throughout the experiment, the mice were monitored for clinical signs of activity, behavior, hair luster, body weight (weighed every 3 days), and general health status. On the 17th day, five mice in each group were sacrificed randomly by cervical dislocation. Then, blood (serum was obtained for serum immunoglobulins), feces, and small intestine were collected under sterile conditions and immediately stored at −80°C. The organs were cleaned with normal saline and weighed to calculate the organ-to-body weight ratio. Fresh small intestinal segments with a length of 1 cm were preserved in 10% (*w/v*) paraformaldehyde (pH 7.0) for further analysis.

### 2.3. Determination of serum immunoglobulins, intestinal sIgA, and intestinal cytokine secretion

Blood was collected and centrifuged at 2,000 rpm for 10 min. Then, serum was retrieved by collecting the supernatants, the serum immunoglobulin levels (IgA, IgG, and IgM) were determined, and the intestinal secretion of sIgA, TGF-β, IFN-γ, TNF-α, IL-6, and IL-18 was measured by using the ELISA kit (Meimian, China) according to the corresponding instructions.

### 2.4. Histological examination

Small intestine tissues (jejunum, duodenum, and ileum) were fixed in 4% paraformaldehyde for 24 h, rinsed in running tap water for 30 min, dehydrated, and embedded in paraffin. The resulting blocks were sliced into 5-μm-thick sections by using a microtome (RM2235, Leica Biosystems, Germany). Finally, the slices were subjected to hematoxylin–eosin (H&E) staining (Xie et al., 2019). The morphological structure of the small intestine segments, including intestinal mucosal thickness, villus height, and crypt depth, was evaluated by observing at least 10 different regions in each section using a Leica DM500 microscope (Leica Microsystem, Germany). The images were analyzed using a quantitative digital image analysis system (Image-Pro Plus 6.0).

### 2.5. 16S rRNA high-throughput sequencing and bioinformatics analysis

Microbial DNA was extracted from 12 fecal samples of mice by using QIAamp Fast DNA Stool Mini Kit (Qiagen, Hilden, Germany) as per the manufacturer’s recommendations. The concentration and the quality of DNA were detected using a nucleic acid detector (Nanodrop, Thermo Scientific NC2000, USA) and 1.2% agarose gel electrophoresis, respectively. Standard bacteria V3–V4 hypervariable region gene PCR primers (forward primer: ACT CCT ACG GGA GGC AGCA; reverse primer: GGA CTA CHV GGG TWT CTA AT) were used. After PCR amplification, the products were subjected to agarose gel electrophoresis, and target gene fragments were recovered by using the AxyPrep DNA Gel Extraction Kit (Axygen, CA, USA). Moreover, TruSeq Nano DNA Low Throughput Library Prep Kit (Illumina, CA, USA) was employed for sequence library construction. The quality of the prepared libraries was checked using the Agilent Bioanalyzer 2100 (Agilent) with Agilent High Sensitivity DNA Kit (Agilent Technologies). The libraries were quantified using Quant-iT PicoGreen dsDNA Assay Kit on the Promega QuantiFluor fluorescence quantification system. The qualified library was sequenced using the MiSeq Reagent Kit V3 (600 cycles) for 2 × 300-bp paired-end reads on an Illumina MiSeq Sequencer.

Sequence analysis was established as operational taxonomic units (OTUs) *via* Uclust with over 97% similarity (Bokulich et al., 2013), and the highest abundance sequence in each OTU was selected as the representative sequence (Caporaso et al., 2010). Then, OTUs were taxonomically classiffed and grouped by comparing with those in the Unite database (Kõljalg et al., 2013). Six metrics were used to analyze the alpha diversity, including Chao, Good’s coverage, Shannon, Simpson, Pielou’s_e, and Observed species. Beta diversity on the weighted unifrac was calculated by using the QIIME software (version 1.7.0), while cluster analysis was preceded by principal coordinate analysis (PCoA) (Ramette, 2007). LEfSe was used to analyze the discrepancy in microbial communities between groups. Statistical analysis was created *via* R software (v3.0.3), and all the data were evaluated statistically by one-way analysis of variance using SPSS 17.0 software (SPSS Inc., Chicago, IL, USA). The original sequence data can be obtained at National Center for Biotechnology Information with accession number PRJNA880707.

### 2.6. Statistical analysis

Statistical analysis of the data for multiple comparisons was performed by one-way analysis of variance followed by Duncan’s test. A level of *P <*0.05 was considered statistically significant.

## 3. Results

### 3.1. Effect of TB on clinical signs and organ-to-body-weight ratio

**Figure 1:**
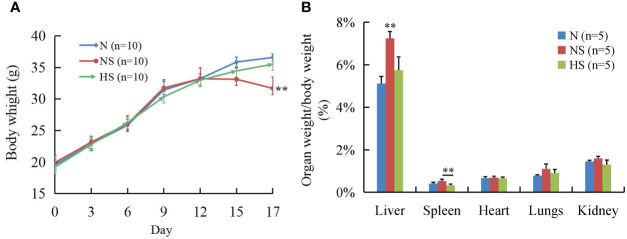
Effect of *T. bellirica (Gaertn.) Roxb.* extract administration on body weight and organ-to-body-weight ratio after *Salmonella Typhimurium* challenge in mice. **(A)** Body weight. **(B)** Organ-to-body-weight ratio. The results were evaluated through one-way ANOVA. All of the data represent means ± SD. Unmarked data indicate no significant difference (*P* > 0.05); **P < 0.01.

In the first 13 days, no significant changes were observed in the clinical signs and behavioral patterns of all mice. At 3 days after the *S. Typhimurium* challenge, the NS group had tremors, reduced activity, shaggy hair, and closed eyes and huddled together compared with the N and HS groups; the body weight significantly decreased in the NS group as well (*P* < 0.01). It is worth noting that non-significant clinical signs and body weight were observed between the N and HS groups (**Figure 1A**). Additionally, the liver/body weight ratio in the NS group was significantly higher than that of the N and HS groups at a probability value of 0.01, while the spleen/body weight ratio was also significantly higher than that in the HS group (*P* < 0.01) (**Figure 1B**).

### 3.2. Effect of TB on serum immunoglobulins, intestinal sIgA, and intestinal cytokine secretion

**Figure 2:**
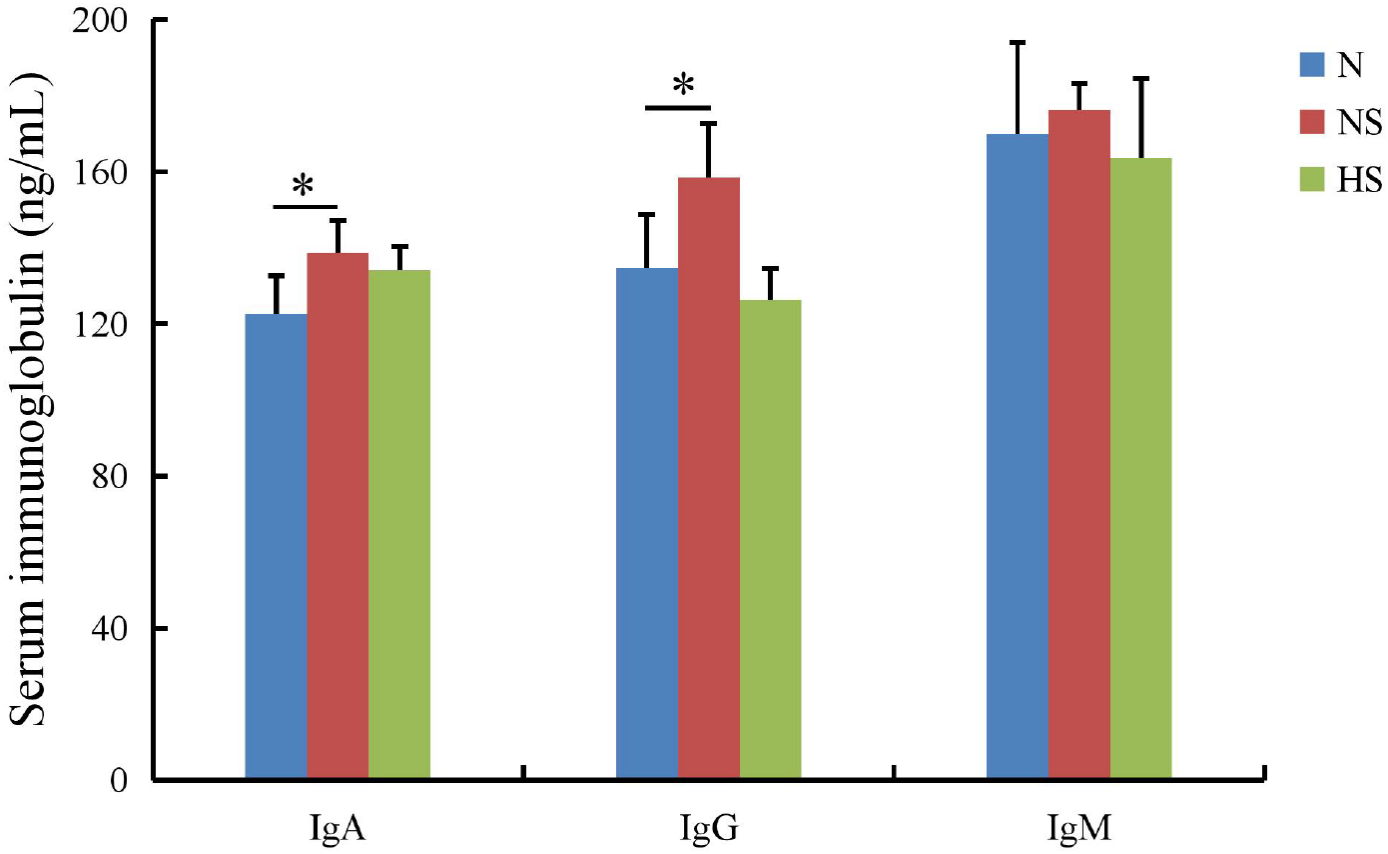
Effect of *Terminalia bellirica (Gaertn.) Roxb.* on serum immunoglobulins in *Salmonella Typhimurium*-infected mice. The results were evaluated through one-way ANOVA. All the data represent means ± SD. Unmarked data indicate no significant difference (*P* > 0.05); *P < 0.05.

**Figure 3:**
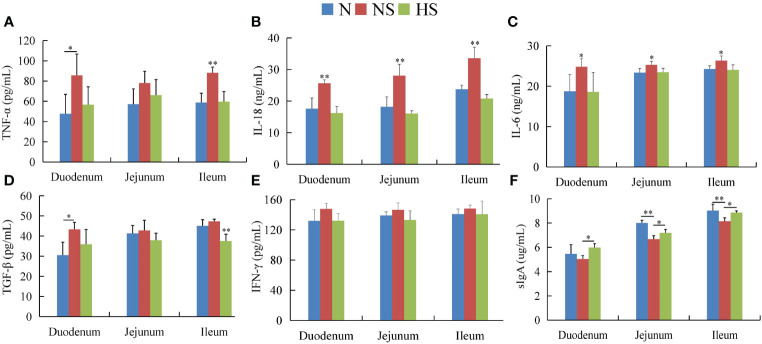
Effect of *Terminalia bellirica (Gaertn.) Roxb.* on intestinal cytokine secretion and intestinal sIgA in *Salmonella Typhimurium*-infected mice. **(A)** TNF-α, **(B)** IL-18, **(C)** IL-6, **(D)** TGF-β, **(E)** IFN-r, and **(F)** sIgA. The results were evaluated through one-way ANOVA. All the data represent means ± SD. Unmarked data indicate no significant difference (*P* > 0.05); *P < 0.05; **P < 0.01.

The serum immunoglobulins showed that the IgA, IgG, and IgM levels in the HS group were not significant compared with those in the N group (*P* > 0.05), while the IgA and IgG levels were remarkably lower than those in the NS group (*P* < 0.05) (**Figure 2**). In the duodenum, the levels of five cytokines were downregulated in the HS group compared with those in the NS group, in which IL-18 (*P* < 0.01) and IL-6 (*P* < 0.05) had more significant differences. In the jejunum, IL-18 (*P* < 0.01) and IL-6 (*P* < 0.05) in the HS group were significantly lower than those in the NS group, while sIgA in the HS group was higher than that in the NS group (*P* < 0.05). Moreover, the level of IL-18 (*P* < 0.01), IL-6 (*P* < 0.05), TNF-α (*P* < 0.01), and TGF-β (*P* < 0.01) in the NS group was significantly higher than that in the N and HS groups in the ileum. Interestingly, the levels of IFN-γ in the HS group had downward trends. Nevertheless, no statistical correlation was observed compared with the NS group. Therefore, the HS group increased the expression of sIgA in the duodenum, jejunum, and ileum (**Figure 3**).

### 3.3. Effect of TB on intestinal physical barrier function

**Figure 4:**
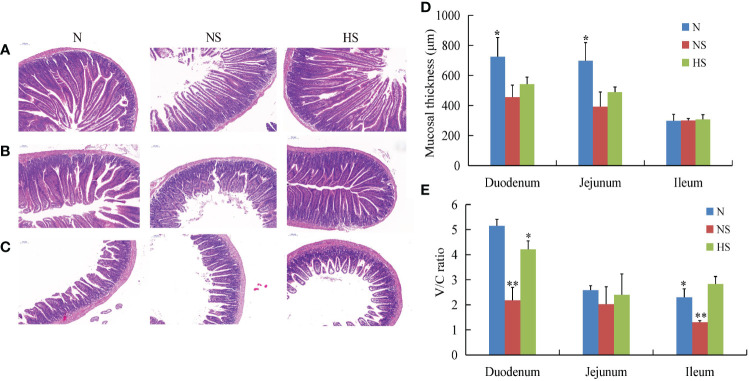
Effect of *Terminalia bellirica (Gaertn.) Roxb.* on intestinal physical barrier function in *Salmonella Typhimurium*-infected mice. **(A–C)** Histological examination of the duodenum, jejunum, and ileum stained with H&E on a 100-μm scale bar. **(D)** Mucosal thickness of intestinal segments. **(E)** Ratio of small intestinal villus height to crypt depth. The results were evaluated through one-way ANOVA. All the data represent means ± SD. Unmarked data indicate no significant difference (*P* > 0.05); *P < 0.05; **P < 0.01.

H&E staining showed a relatively great change in the morphological structures of the intestinal mucosa that occurred in different groups. The intestinal damage was mainly characterized by the loosening of the lamina propria, erosion of the villi, and loss of goblet cells. This study’s results show that the intestines of the NS group were more damaged than those in the other groups (**Figures 4A–C**). Furthermore, the mucosal thickness in the HS group was higher than that in the NS group. However, no obvious difference was observed between the two groups (*P* > 0.05) (**Figure 4D**). In addition, the HS group exhibited tidily and tightly arranged columnar epithelial cells with a significant increase in villus height/crypt depth ratio (V/C ratio) when compared with the NS group (*P* < 0.05) (**Figure 4E**).

### 3.4. Effect of TB on the intestinal microbiota composition

**Figure 5:**
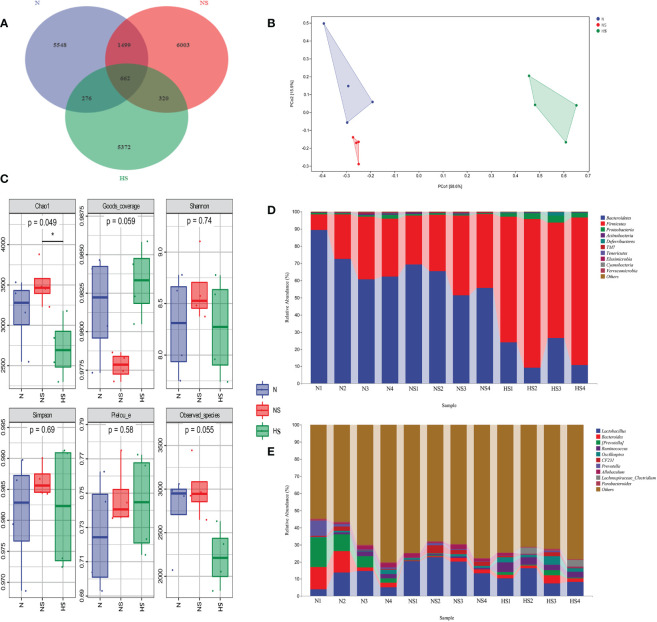
Effect of *Terminalia bellirica (Gaertn.) Roxb.* on intestinal microbiota composition in *Salmonella Typhimurium*-challenged mice. **(A)** Fecal microflora of mice analyzed by a Veen diagram. Relative abundance of gut bacterial taxa among different groups. **(B)** Principal component analysis (PCA) of the fecal microbiota; PCA map based on Euclidean distance. Each point indicates one sample; the distance of the two points indicates the difference in fecal microbiota. **(C)** Diversity indices of the fecal microbiota in different groups; Chao1, Goods_Coverage, Shannon, Simpson, Pielou’s_e, and Observed indices were used to evaluate the alpha diversity of the fecal microbiota. **(D)** Phylum level. **(E)** Genus level. The results were evaluated through one-way ANOVA. All the data represent means ± SD. *P < 0.05.

The current study subjected 12 fecal samples collected from mice to high-throughput sequencing analysis. After optimizing the preliminary data, 214,626, 212,107, and 254,684 high-quality valid sequences were obtained from the N, NS, and HS groups, respectively. Subsequently, the high-quality sequences were merged, and OTU was partitioned based on 97% nucleotide sequence similarity. Moreover, the Veen map/diagram analysis demonstrated that 662 bacterial species were shared among the three groups. The HS group showed 5,372 common bacteria species, which were not found in the other groups (**Figure 5A**). In terms of the alpha diversity of intestinal microbiota, no obvious difference was observed in the Goods_coverage, Shannon, Simpson, Pielou’s_e, and Observed_species among different groups. The statistical analysis showed that both the NS and HS groups exhibited the Chao1 significant differences (*P* < 0.05), indicating that *S. typhimurium* and TB changed the richness and evenness of intestinal flora. It is exceptionally notable that significant differences were found in the microbial community structure by PCoA in different groups, especially in the HS group, compared with the two other groups (**Figures 5B, C**).

According to the taxonomic composition analysis, it was determined that Bacteroidetes (71.2 ± 11.43% in the N group, 60.34 ± 7.22% in the NS group, and 17.58 ± 7.66% in the HS group) and Firmicutes (26.27 ± 10.58% in the N group, 37.77 ± 7.30% in the NS group, and 78.16 ± 8.23% in the HS group) were dominant in all samples at the phylum level (**Figure 5D**). Other phyla, including Proteobacteria, Actinobacteria, and Deferribacteres, presented a lower abundance (<5% of all samples) (**Figure 5D**). *Lactobacillus*, *Bacteroides*, *Prevotella*, *Oscillospira*, *CF231*, and *Ruminococcus* were likewise predominant bacterial genera (**Figure 5E**).

**Figure 6:**
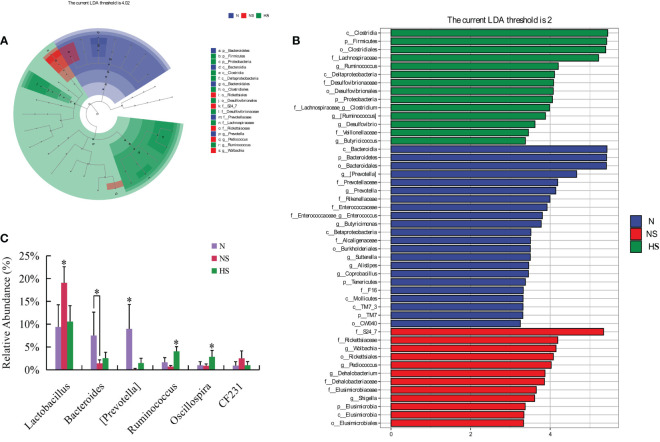
**(A)** LEfSe taxonomic cladogram. The colored nodes from the inner circle to the outer circle represented the hierarchical relationship of all taxa from the phylum to the genus level. Taxa enriched in HS, NS, and N group are shown in green, red, and blue, respectively. The non-significant changes were colored white. The diameter of each small circle represents the taxa abundance. **(B)** Enriched taxa with a linear discriminant analysis score = 2 are shown in the histogram. The longer bars of the histogram represent the more significant phylotype microbiota upon comparison. **(C)** Species with significant differences at the genus level. Unmarked data indicate no significant difference (*P* > 0.05); *P < 0.05; *n* = 4.

We performed LEfSe analysis among the N, NS, and HS groups to identify the key phylotypes of gut microbiota in different groups. An evolutionary clustering analysis diagram was delivered to identify major microflora by taxonomy (**Figure 6A**). In the cladogram, Firmicutes, Proteobacteria, and Clostridia had the highest flora abundance in the green parts. Rickettsiales was the richest in the red area, and Bacteroidetes was the richest in the blue area, representing the HS, NS, and N groups. Then, the linear discriminant analysis (LDA) results showed 22, 12, and 14 discriminative features in the N, NS, and HS groups, respectively (LDA = 2, *P* < 0.05) (**Figure 6B**). Overall, these results indicated that TB prevention altered the key phylotypes of the gut microbiota and promoted the multiplication of specific bacteria in *S. Typhimurium*-challenged mice.

The abundance of dominant fecal bacteria was analyzed among the three groups. The relative abundance of *Ruminococcus* and *Oscillospira* in the HS group was significantly enhanced compared with those of the other groups (*P* < 0.05) at the genus level. Similarly, the abundance of *Lactobacillus* and *Bacteroides* was not significant compared with those in the N and HS groups (*P >* 0.05) (**Figure 6C**).

## 4. Discussion

At present, the treatment of *Salmonella* is mainly based on antibiotics (Sengupta et al., 2013). Conversely, antibiotic treatment can promote cooperative virulence within host evolution by increasing the duration of transmissibility. Thereby, it enhances the spread of an infectious disease (Diard et al., 2014; Stapels et al., 2018). Alternatively, antimicrobial resistance poses a worldwide threat to human health and biosecurity (Yu et al., 2021). Thus, a substance that could attenuate the virulence and pathogenicity of bacteria could more effectively remove the bacteria without a breakthrough in the fight against bacterial resistance (Lupien et al., 2015; Sun et al., 2019). Traditional Chinese medicine has been proven effective in the treatment of diseases (Chu et al., 2020). These plant-derived bioactive compounds improve growth performance, immunity, and intestinal health in animal husbandry (Wu, 2018; Long et al., 2021). However, the effect on growth performance, immunity, intestinal mucosal barrier, and intestinal microbiota remains to be determined in *S. Typhimurium*-infected mice. Our results suggest a general principle in which TB can regulate immunity in many ways.

In previous studies, some herbs have been used for their anti-inflammatory activity in different disease conditions, *e*.*g*., diabetes and tumor with enhanced growth performance (Chuengsamarn et al., 2012; He et al., 2018; Wang et al., 2020; Li et al., 2021c). The fruits of TB elicit various pharmacological properties, such as antioxidant, antidiabetic, analgesic, antidiarrheal, and anti-inflammatory effects (Jayesh et al., 2019). The vessels’ permeability might increase in *Salmonella-*infected mice, causing dissemination to the liver and spleen (Langer et al., 2019) and, subsequently, an increase in proliferative tissue lesions (Larson and Bell, 1915). Interestingly, our results showed that no significant difference was observed in the organ-to-body-weight ratio of TB and vehicle control groups. These results are similar to those of a previously reported compound with antimicrobial properties (Kwon et al., 2008). Unfortunately, this paper does not investigate the bacterial load in the organs or feces of the different experimental groups after the *S. Typhimurium* challenge. This is a shortcoming of the present study, which warrants further investigation.

The intestine is a location where nutrients are digested and absorbed in animals as well as the first protective barrier involved in dealing with harmful or toxic substances that enter the body. We consistently quantified intestinal epithelial renewal and structural integrity in terms of villus height, crypt depth, and cell number, thereby indirectly affecting health and body functions (Ma et al., 2020). Our results suggested that the V/C ratio of TB extract significantly increased. The gavage remarkably decelerated the pathological damage, and normal crypt depth was maintained to avoid crypt hyperplasia in mice. Thus, our results conveyed important information that TB may strengthen the intestinal physical barrier function and enhance the ability to defend against pathogenic microorganisms, which was similar to the findings of the study by Xie et al. (2019) and Li et al. (2021b).

Immunoglobulins are proteins of the same category, with their activity and chemical structure being similar to those of antibodies. The IgG, IgA, and IgM levels can reflect the strength of humoral immunity in serum (Patel et al., 2010). Serum immunoglobulin levels were slightly increased with *Salmonella* infection (Campos-Rodríguez et al., 2006). The supplementation of traditional Chinese medicine can improve both cellular immunity and humoral immunity. The results of the present study showed that the IgA and IgG levels of mice supplemented with TB were lower than those of the NS group, possibly because of the involvement of TB in defense against *S. Typhimurium* infection. Immunoglobulin A (IgA) is a major class of antibodies that is secreted by the intestinal mucosa. It contributes to intestinal barrier function (Planer et al., 2016) and passive immunization of sIgA for combating enteric diseases (Richards et al., 2021). Remarkably, polysaccharides could promote sIgA from many traditional medicines (Zou et al., 2022b). TB can likewise increase intestinal sIgA secretion to a certain extent. These results were uniformly consistent with the results of a previous study that expounded on cranberry proanthocyanins (Pierre et al., 2014) and taxifolin (Hou et al., 2020) as supplements. They have an increasing effect on the sIgA level to alleviate intestinal inflammation. Moreover, considering that inflammation is a balance between the pro-inflammatory and anti-inflammatory cytokines, this balance tends to shift towards the pro-inflammatory cytokines during inflammation, and subsequently the exuberant local cytokine levels further result in a systemic cytokine storm and inflammation (Dalmasso et al., 2008). Usually, the intestinal mucosa is damaged by pathogenic microorganisms that trigger submucosal lamina propria macrophages and T lymphocytes with the secretion of large amounts of cytokines, including IL-1, IL-6, IL-17, IL-23, and TNF-α (Su et al., 2009). Several studies have linked traditional medicines to the downregulation of inflammatory factors such as TNF-α, IL-1B, IL-18, and IL-6 (Zheng et al., 2012; Ha et al., 2021; Dai et al., 2021). We observed that TB therapy reduced the intestinal inflammatory factors TGF-ß, IFN-γ, TNF-α, IL-6, and IL-18 in *Salmonella*-infected mice. Furthermore, it effectively regulates immunity to decrease the cytokine storm, which is consistent with the results of previous studies (Jayesh et al, 2017b). The gut barrier disruption may elicit persistent immune activation in the host (Costa et al., 2019). Thus, up to now, we hypothesize that a role in the oral administration of TB may strengthen the association between the physical barrier and immunological barrier to regulate intestinal barrier function.

The intestinal microbiota constitutes a complex microbial ecosystem, each of which may have distinct functional roles, thus affecting the development, immunological responses, and nutritional status of the host (Li et al., 2019a). The imbalance of gut flora may especially result in the disruption of the gut barrier which will cause multiple diseases (Citi, 2018). *S. Typhimurium*, a gram-negative pathogen, induces impairment in intestinal microbiota dysbiosis and metabolism disorder (Stecher et al., 2007). Admittedly, several studies have proven that the gut microbiota is significantly regulated after taking a certain dose of Chinese medicine, which greatly increased the relative abundance of *Lactobacillus* for the treatment of constipation (Li et al., 2020), and siwu-yin may improve the composition of the intestinal flora to inhibit the occurrence of precancerous esophageal lesions (Shi et al., 2022). Dextran sulfate sodium-induced changes in colon microbiota composition and microbial functions are regulated by taxifolin (Hou et al., 2020). Moreover, 16S rRNA sequencing revealed that *S. Typhimurium* and TB change the gut microbial community composition and diversity. Our results indicated that Bacteroidetes, Firmicutes, Proteobacteria, and Actinobacteria were the most predominant phyla in three groups of mice. Interestingly, the Firmicutes level in the HS group had a sharply increasing trend compared with the other groups, which was consistent with previous observations (Chen et al., 2021). Generally, Firmicutes is among the largest phyla, and the dietary fiber–Firmicutes–host axis has manifested the beneficial effects of dietary fiber-induced Firmicutes and their metabolites on health (Sun et al, 2022) Notably, Deferribacteres was gradually enhanced in the H group compared with the other groups. Remarkably, Deferribacteres could gain energy through obligate or facultative anaerobic metabolism, thus altering the expression of iron-metabolizing proteins that further increase iron intake (Li et al., 2019b). On the other hand, iron absorption was reduced during enteritis (Naveh et al., 2000). However, the supplement lactoferrin has prevented intestinal inflammation (Fan et al., 2022), emphasizing a role for Deferribacteres. In comparison with the N group, Bacteroidetes and Actinobacteria proportions were lower in the intestinal microbiota in the mice of the TB group, which was consistent with the results of a previous study (Yan et al., 2020). Research has shown that Bacteroidetes is typical of most gram-negative bacilli; for example, *B. fragilis* secretes an unusually complex mixture of neurotoxins, including the extremely pro-inflammatory lipopolysaccharide BF-LPS (Zhao and Lukiw, 2018). Actinobacteria produce most of the clinically used antibiotics and many other natural products with medical or agricultural applications (van Bergeijk et al., 2020). However, many bacteria are potentially pathogenic (Zalar et al., 2022), which can easily translate into pathogenic interactions with the host (Miao and Davies, 2010). Therefore, our results conveyed the important information that the structure of the gut microbiota may change and increase the abundance of beneficial bacteria at the phyla level by TB. Compared with the N and NS groups, the HS group had higher *Ruminococcus* and *Oscillospira* levels at the genus level. Remarkably, *Ruminococcus* forms a multi-enzyme cellulosome complex that could play an integral role in the ability of this bacterium to degrade plant cell wall polysaccharides (Berg Miller et al., 2009). In particular, *Oscillospira* exhibits positive regulatory effects in areas related to obesity and chronic inflammation and can be developed as the next generation of probiotics (Yang et al., 2021). The other dominant genera had no significant change between the N and HS groups. A phenomenon may be proposed such that the normal gut flora was maintained by TB and guarded and increased the abundance of beneficial bacteria. Furthermore, the intestinal microbiota forms a biofilm and promotes the differentiation of intestinal epithelial cells against pathogenic microorganisms (Li et al., 2021a), which is perhaps the main mechanism in protecting mice from invasive *S. Typhimurium* infections.

In summary, these results support the hypothesis that the administration of TB can protect against *S. Typhimurium* by alleviating the immunological barriers and normalizing the gut microbiota. Our findings highlight a promising application of TB in the prevention of *S. Typhimurium* infections. Thus, our results provide new insights into the biological functions of TB for the preventive effect of intestinal inflammation.

## Data availability statement

The datasets presented in this study can be found in online repositories. The names of the repository/repositories and accession number(s) can be found below: https://www.ncbi.nlm.nih.gov/ , PRJNA880707.

## Ethics statement

The study protocol was approved by the Committee for Animal Research of Tibet Agricultural & Animal Husbandry University, China (Unified social credit code: 12540000MB0P013721).

## Author contributions

QK: conceptualization and writing of the original draft. ZS: methodology. YL, ZT, and YX: formal analysis and investigation. MK: review and editing. JL and SL: supervision, technical assistance, and funding. All authors participated in the writing of the manuscript, read, and approved the final manuscript.
